# Minimally Invasive Complete Response Assessment of the Breast After Neoadjuvant Systemic Therapy for Early Breast Cancer (MICRA trial): Interim Analysis of a Multicenter Observational Cohort Study

**DOI:** 10.1245/s10434-020-09273-0

**Published:** 2020-12-02

**Authors:** Ariane A. van Loevezijn, Marieke E.M. van der Noordaa, Erik D. van Werkhoven, Claudette E. Loo, Gonneke A. O. Winter-Warnars, Terry Wiersma, Koen K. van de Vijver, Emilie J. Groen, Charlotte F. J. M. Blanken-Peeters, Bas J.G.L. Zonneveld, Gabe S. Sonke, Frederieke H. van Duijnhoven, Marie-Jeanne T. F. D. Vrancken Peeters

**Affiliations:** 1 Departments of Surgical Oncology, Netherlands Cancer Institute - Antoni van Leeuwenhoek, Plesmanlaan 121, 1066 CX Amsterdam, The Netherlands; 2 Biometrics, Netherlands Cancer Institute - Antoni van Leeuwenhoek, Amsterdam, The Netherlands; 3 Radiology, Netherlands Cancer Institute - Antoni van Leeuwenhoek, Amsterdam, The Netherlands; 4 Radiation Oncology, Netherlands Cancer Institute - Antoni van Leeuwenhoek, Amsterdam, The Netherlands; 5 Department of Pathology, Ghent University Hospital, Ghent, Belgium; 6 Pathology, Netherlands Cancer Institute - Antoni van Leeuwenhoek, Amsterdam, The Netherlands; 7 Department of Surgical Oncology, Rijnstate Hospital, Arnhem, The Netherlands; 8 Department of Radiology, Deventer hospital, Deventer, The Netherlands; 9 Medical Oncology, Netherlands Cancer Institute - Antoni van Leeuwenhoek, Amsterdam, The Netherlands

## Abstract

**Background:**

The added value of surgery in breast cancer patients with pathological complete response (pCR) after neoadjuvant systemic therapy (NST) is uncertain. The accuracy of imaging identifying pCR for omission of surgery, however, is insufficient. We investigated the accuracy of ultrasound-guided biopsies identifying breast pCR (ypT0) after NST in patients with radiological partial (rPR) or complete response (rCR) on MRI.

**Methods:**

We performed a multicenter, prospective single-arm study in three Dutch hospitals. Patients with T1–4(N0 or N +) breast cancer with MRI rPR and enhancement ≤ 2.0 cm or MRI rCR after NST were enrolled. Eight ultrasound-guided 14-G core biopsies were obtained in the operating room before surgery close to the marker placed centrally in the tumor area at diagnosis (no attempt was made to remove the marker), and compared with the surgical specimen of the breast. Primary outcome was the false-negative rate (FNR).

**Results:**

Between April 2016 and June 2019, 202 patients fulfilled eligibility criteria. Pre-surgical biopsies were obtained in 167 patients, of whom 136 had rCR and 31 had rPR on MRI. Forty-three (26%) tumors were hormone receptor (HR)-positive/HER2-negative, 64 (38%) were HER2-positive, and 60 (36%) were triple-negative. Eighty-nine patients had pCR (53%; 95% CI 45–61) and 78 had residual disease. Biopsies were false-negative in 29 (37%; 95% CI 27–49) of 78 patients. The multivariable associated with false-negative biopsies was rCR (FNR 47%; OR 9.81, 95% CI 1.72–55.89; *p *= 0.01); a trend was observed for HR-negative tumors (FNR 71% in HER2-positive and 55% in triple-negative tumors; OR 4.55, 95% CI 0.95–21.73; *p *= 0.058) and smaller pathological lesions (6 mm vs 15 mm; OR 0.93, 95% CI 0.87–1.00; *p *= 0.051).

**Conclusion:**

The MICRA trial showed that ultrasound-guided core biopsies are not accurate enough to identify breast pCR in patients with good response on MRI after NST. Therefore, breast surgery cannot safely be omitted relying on the results of core biopsies in these patients.

**Electronic supplementary material:**

The online version of this article (10.1245/s10434-020-09273-0) contains supplementary material, which is available to authorized users.

## Introduction

With systemic treatments becoming increasingly effective, the number of breast cancer patients undergoing breast conserving surgery after neoadjuvant systemic therapy (NST) has increased, and pathological complete response (pCR) occurs more frequently.^1^^–^3 Previous studies have demonstrated that excision of the residual disease, rather than the entire initial tumor bed, does not compromise the recurrence rate in patients undergoing breast conserving treatment after NST.^4^,^5^ It can thus be questioned as to whether any surgical resection was needed in patients with pCR in the surgical specimen.

A major challenge in pursuing a surgery-free treatment strategy for patients with pCR is the identification of pCR without surgery. Current imaging modalities such as ultrasound, MRI, and^18^F-FDG PET-CT-scan are not sufficiently accurate to identify pCR.^6^,^7^ Minimally invasive biopsies to detect the presence of residual tumor in the breast after NST have been explored in several pilot studies.^8^^–^14 The primary outcome of these studies was the false-negative rate (FNR), defined as the proportion of patients with residual disease in the surgical specimen of the breast that had tumor-negative biopsies after NST. Promising FNRs were achieved in some of these studies, leading to the initiation of new trials with a 10% cut-off for the FNR of biopsies assessing pCR (see supplemental Table).^8^,^9^,^13^,^14^

We designed the MICRA trial (Minimally Invasive Complete Response Assessment of the breast after NST) to determine whether ultrasound-guided core biopsies of the breast are sufficiently accurate to differentiate between breast pCR and residual disease (irrespective of nodal status) in patients with a radiological complete or partial response on MRI.^15^ Here, we present the results of the interim analysis.

## Methods

### Study Design and Participants

This multicenter, prospective, single-arm study included women aged 18 years or older with stage I–III invasive breast cancer of any subtype receiving NST. Key eligibility criteria were placement of a marker centrally in the tumor before the start of NST and a radiological complete (rCR) or partial response (rPR, residual size ≤ 2.0 cm and ≥ 30% decrease in tumor size) on dynamic contrast-enhanced (DCE)-MRI after NST according to RECIST criteria.^16^ Exclusion criteria were histopathologically confirmed DCIS before the start of NST and a history of ipsilateral breast surgery and/or radiotherapy. Patients were enrolled in three Dutch hospitals (the Netherlands Cancer Institute, Deventer Hospital, and Rijnstate Hospital). The medical ethical committee of the Netherlands Cancer Institute approved the conduct of the study.

### Procedures

Mammography, ultrasound, and DCE-MRI were used for assessment of the primary tumor and axillary nodes prior to NST. Core needle biopsies (14 G) from the primary tumor were obtained to determine breast cancer subtype and grade (according to the modified Bloom-Richardson system) and fine needle aspiration (FNA) was performed of suspect lymph nodes. Estrogen receptor and progesterone receptor were defined as positive if expression was ≥ 10%, and immunohistochemistry assessment of HER2 overexpression was regarded as positive if 3 + or 2 + with positive in situ hybridization, according to ASCO-CAP guidelines. Before the start of NST, the breast lesion was localized with a marker (e.g., iodine seed, clip, hydromarker, twist marker) followed by mammography and/or ultrasound to confirm adequate position of the marker.

**Fig. 1:**
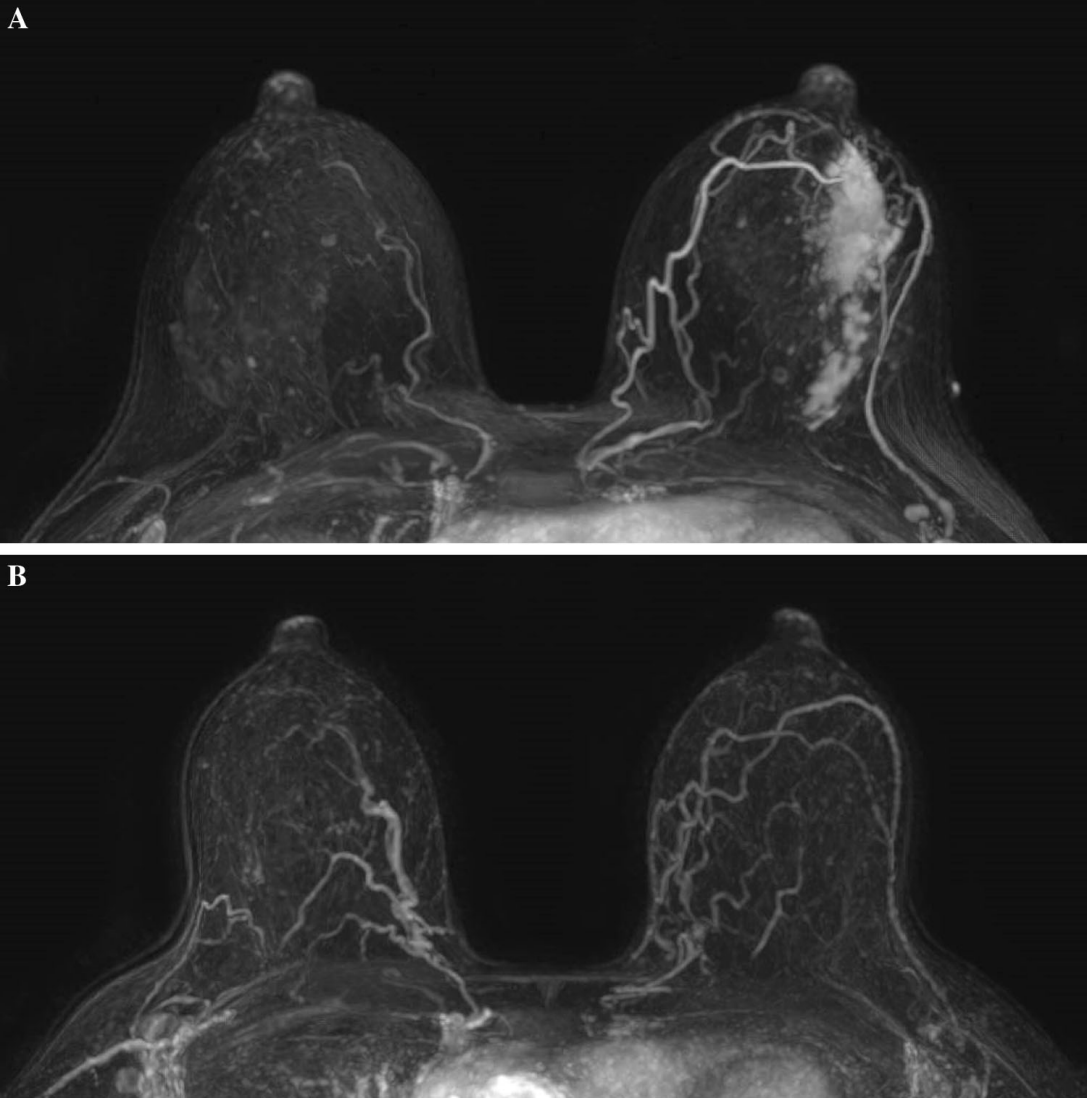
Radiological complete response on dynamic contrast-enhanced MRI after neoadjuvant systemic therapy. Breast MRI in a patient with left-sided breast cancer before the start of neoadjuvant systemic therapy (**A**) and after neoadjuvant systemic therapy (**B**). Maximum intensity projection (MIP) images after treatment show no pathologic enhancement in the left breast, radiologically assessed as a complete response

Patients with hormone receptor (HR)-positive/HER2-negative tumors were treated with four cycles of two-weekly cyclophosphamide and doxorubicin, followed by 12 weekly administrations of paclitaxel. Patients with triple-negative tumors in addition received carboplatin concurrent with paclitaxel. Patients with HER2-positive tumors received nine cycles of paclitaxel, carboplatin, trastuzumab, and pertuzumab (PTC-Ptz), or three cycles of 5-fluorouracil, epirubicin, cyclophosphamide, trastuzumab, and pertuzumab (FEC-T-Ptz), followed by six cycles PTC-Ptz.^2^ Patients with cT1N0 HER2-positive disease received twelve weekly cycles of paclitaxel and trastuzumab. All patients underwent DCE-MRI before the start and at the end of NST with a 1.5-T system (in 18 patients, GE healthcare, Eindhoven, the Netherlands) and a 3.0-T system (in 201 patients, Philips Medical Systems, Best, the Netherlands) using dedicated phased array bilateral breast coils. Images were acquired in the axial plane with the patient in prone position. The MRI protocol consists of a DCE T1-weighted sequence, a diffusion-weighted sequence, and optionally a fast dynamic sequence as previously described.^15^ MRI examinations were assessed by breast radiologists. Radiological complete response (rCR) was defined as complete absence of pathological (i.e., non-physiological) contrast enhancement in the original tumor area. Radiological partial response was defined as 0.1–2.0 cm contrast enhancement and ≥ 30% decrease in tumor size, according to RECIST 1.1 criteria^16^ (Fig. 1). Other radiologic features analyzed were presence of non-mass enhancement and multifocality on MRI, and presence of calcifications on mammography.

Biopsies and the surgical procedure were performed within 6 weeks after NST. Specialized breast radiologists obtained a maximum of eight ultrasound-guided biopsies of the initial tumor area with a 14-gauge (14-G) automated needle device and a 22-mm-throw biopsy gun (Bard Magnum biopsy Instrument, Covington, GA, USA), concentrically around a pre-NST placed marker: four central biopsies close to the marker, and four more peripheral biopsies. In patients with multifocal or multicentric tumors, more than one marker may be used to facilitate breast conserving surgery in patients with good NST response. In these patients, biopsies were obtained from the index lesion or from the largest marked residual lesion, and compared with pathology analysis of this lesion only. To minimize patient discomfort, all biopsies were performed in the operating room under general anesthesia. The surgical procedure was performed immediately thereafter. Breast and axillary surgery were left to the discretion of the institute.

### Outcomes

The primary outcome of the MICRA trial was the FNR of the biopsy procedure, i.e., the proportion of patients with residual disease in the surgical specimen of the breast in whom the biopsies were tumor-negative. Histopathological analyses of the biopsies were categorized as (1) histopathologically representative, containing residual tumor cells or signs of the former tumor bed, (2) unknown, containing normal breast, fatty, or connective tissue, and (3) non-representative, containing small non-assessable tissue.^15^ A pathological complete response (pCR) was defined as absence of invasive and in situ carcinoma in the breast, irrespective of nodal status (ypT0). Response of the breast was assessed according to the Pinder classification system.^17^,^18^

Secondary outcome measures were specificity, sensitivity, positive predictive value, and negative predictive value of the biopsy procedure. In addition, patient, tumor, and imaging characteristics were collected to evaluate correlations with a false-negative outcome.

### Statistical Analysis

We hypothesized that the true FNR was 3%. The null hypothesis was a FNR of 8%. It was calculated that 130 patients with residual disease in the surgical specimen were sufficient to show, with 80% power, that the FNR would not exceed 8% using a one-sided binomial test with a significance α-level of 0.05. Based on published data, a pCR rate of 65% is expected among patients with a rCR and a pCR rate of 12% among patients with a rPR.^7^,^19^ Therefore, 375 patients with rCR and 150 patients with rPR would be required. Taking into account an approximate 10% biopsy failure rate due to technical difficulties, we required inclusion of 575 patients at final analysis.^15^ An interim analysis for futility was planned after inclusion of 150 patients with rCR on MRI.

The two-sided 95% confidence intervals for the FNR and for proportions of patients with pCR were calculated using the Clopper-Pearson exact method. Patients in whom biopsies could not be obtained were excluded from analysis.

Differences between patients with false-negative and true-positive biopsies were tested using the Kruskal–Wallis rank sum test, Fisher’s exact test, and Pearson’s Chi squared test. Subgroup analyses were prespecified for histopathological classification, Bloom-Richardson grade, hormone receptor status, tumor size on MRI, presence of non-mass enhancement or multifocality on MRI, presence of microcalcifications on mammography, and clinical tumor and nodal stage. Post-hoc analyses, including size of the residual lesions at pathology analysis, were also performed. Logistic regression was used to identify factors associated with a false negative result. Statistical significance for comparisons between groups was defined as *p* < 0.05. The conditional power calculations were performed with PASS software version 15.0.4. All other statistical analyses were done using R (version 3.5.0). This study is registered with the Netherlands Trial Register, number NTR6120.

## Results

### Study Participants

**Fig. 2:**
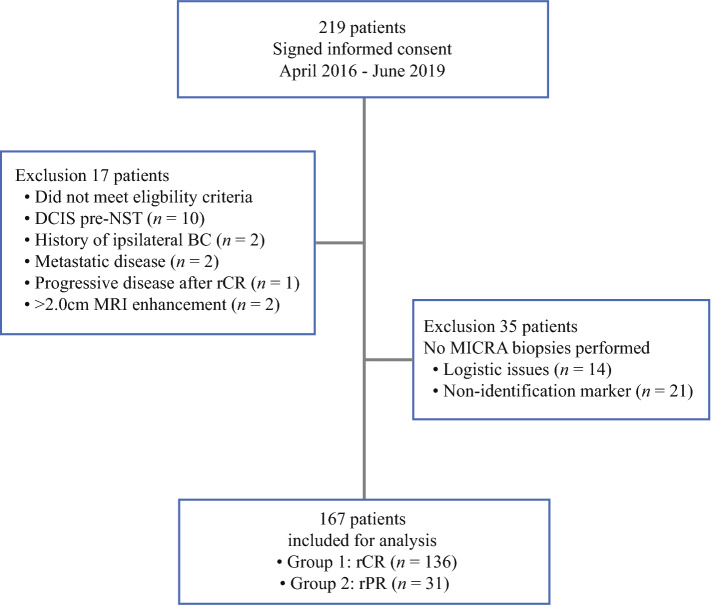
Flowchart. Patient inclusion at interim analysis. *rCR*, radiological complete response; *rPR*, radiological partial response; *NST*, neoadjuvant systemic therapy; *BC*, breast cancer

Between April 2016 and June 2019, we enrolled 219 patients, of which 202 patients fulfilled eligibility criteria. Protocol violations were identified in 17 patients, mainly due to missed DCIS in pre-NST obtained diagnostic biopsies. In 35 patients, post-NST biopsies were not performed. This was due to non-identification of the marker in 21 patients, and due to logistic reasons in 14 patients. Thus, a total of 167 (76%) patients were included for interim analysis (Fig. 2).

Median age was 49 years (IQR 42–56). Tumor histology was invasive ductal carcinoma (IDC) in 146 patients, invasive lobular carcinoma in 14 patients, and other special-type carcinomas in 7 patients. Distribution of tumor subtype by hormone receptor and HER2-expression was HR-positive/HER2-negative in 43 (26%) patients, HR-positive/HER2-positive in 41 (24%) patients, HR-negative/HER2-positive in 23 (14%) patients, and triple-negative in 60 (36%) patients. Mean tumor size on DCE-MRI prior to NST was 27 mm (IQR 21–40). Fifty percent (84 of 167; 95% CI 42–58) of patients were clinically node-positive prior to NST. Post-NST MRI showed rCR in 136 of 167 (81%, 95% CI 75–87) patients and rPR in 31 of 167 (19%; 95% CI 13–25) patients. Baseline patient characteristics are listed in Table 1.

**Table 1:** Baseline characteristics by radiological response group

	Complete response MRI	Partial response MRI	Total
	(*n* = 136)	(*n* = 31)	(*n* = 167)
Age	48 (42–56)	50 (43–56)	49 (42–56)
*Clinical tumor stage*			
T1	32 (24%)	4 (13%)	36 (21%)
T2	87 (64%)	20 (65%)	107 (64%)
T3	17 (12%)	6 (19%)	23 (14%)
T4	0	1 (3%)	1 (1%)
*Clinical nodal stage*			
N+	68 (50%)	16 (52%)	84 (50%)
*Imaging features*			
Multifocal	31 (23%)	9 (29%)	40 (24%)
Non-mass	27 (20%)	6 (19%)	33 (20%)
Calcifications	36 (27%)	9 (29%)	45 (27%)
Tumor size (mm)	27 (20–40)	27 (22–40)	27 (21–40)
*Histology*			
Ductal	121 (89%)	25 (81%)	146 (88%)
Lobular	10 (7%)	4 (13%)	14 (8%)
Other	5 (4%)	2 (6%)	7 (4%)
*Tumor subtype*			
HR +/HER2 -	32 (24%)	11 (35%)	43 (26%)
HR +/HER2 +	36 (26%)	5 (16%)	41 (24%)
HR -/HER2 +	21 (15%)	2 (7%)	23 (14%)
Triple-negative	47 (35%)	13 (42%)	60 (36%)
*Tumor grade*			
Grade 1	7 (5%)	0	7 (4%)
Grade 2	41 (30%)	15 (48%)	56 (34%)
Grade 3	80 (59%)	15 (48%)	95 (57%)
Unknown	8 (6%)	1 (3%)	9 (5%)

Data are median (IQR) or *n* (%). All baseline characteristics were assessed before administration of neoadjuvant systemic therapy. Calcifications were assessed on mammography, other imaging features were assessed on MRI

### Pathology Analysis

Post-NST, a median of eight (IQR 8–8) 14-G ultrasound guided biopsies per patient were obtained, followed by breast conserving surgery in 140 (84%) patients and mastectomy in 27 (16%) patients. Biopsies were representative in 151 (90%) patients, not representative in eight (5%) patients, and representativeness was unknown in eight (5%) patients.

In total, 89 (53%, 95% CI 45–61) of 167 patients had pCR in the surgical specimen, while 78 had residual disease. Eighty-one (91%) of the 89 patients with breast pCR had no axillary metastases (ypT0N0). The pCR rate was 60% (81 of 136) in patients with rCR on MRI and 26% (8 of 31) in patients with rPR on MRI (Table 2).

**Table 2:** Pathological response assessment by radiological response group

	Complete response MRI (*n* = 136)	Partial response MRI (*n* = 31)	Total (*n* = 167)
*Pathological response surgical specimen*			
No residual carcinoma (1i)	81 (60%)	8 (26%)	89 (53%)
No residual invasive but DCIS (1ii)	8 (6%)	0	8 (5%)
Minimal residual disease, < 10% (2i)	31 (23%)	8 (25%)	39 (23%)
10-50% of tumor remaining (2ii)	11 (8%)	12 (39%)	23 (14%)
>50% of tumor remaining (2iii)	3 (2%)	3 (10%)	6 (4%)
No evidence of response (3)	1 (1%)	0	1 (1%)
Only LVSI present	1 (1%)	0	1 (1%)
*Pathological response biopsies*			
Tumor-negative	107 (79%)	11 (35%)	118 (71%)
Tumor-positive	29 (21%)	20 (65%)	49 (29%)

Data are *n* (%). *LVSI*, lymphovascular invasion

### The False-Negative Rate of the Biopsy Procedure

In 29 of the 78 patients without pCR in the surgical specimen, the residual disease was not present in the biopsies. Thus, the FNR of the biopsies assessing pCR was 37% (29 of 78; 95% CI 27–49). Sensitivity of the biopsies was 63% (49 of 78, 95% CI 51–74), specificity was 100% (89 of 89, 95% CI 0.96–1), positive predictive value was 100% (49 of 49, 95% CI 0.93–1) and negative predictive value was 75% (89 of 118, 95% CI 67–83) (Table 3). Biopsies had been scored as non-representative in two of 29 patients with false-negative biopsies and representativeness was unknown in four patients.

**Table 3:** False-negative rate of biopsies identifying pathological complete response of the breast

Biopsies	Residual disease in surgical specimen					
	No (*n* = 89)			Yes (*n* = 78)		
	rPR	rCR	Total	rPR	rCR	Total
Tumor-neg	8 (9%)	81 (91%)	89 (100%)	3 (4%)	26 (33%)	29 (37%)
Tumor-pos	0	0	0	20 (26%)	29 (37%)	49 (63%)
Total	8 (9%)	81 (91%)	89 (100%)	23 (29%)	55 (71%)	78 100%)

Data are *n* (%). *rCR*, radiologic complete response on MRI. *rPR*, radiologic partial response on MRI

The FNR differed per response group and tumor subtype. In the rCR group, the FNR was 47% (26 of 55; 95% CI 34–61) and in the rPR group, the FNR was 13% (3 of 23; 95% CI 3–34) (*p* = 0.005). The FNR was 24% (8 of 34; 95% CI 11–41) in HR-positive/HER2-negative tumors, 29% (5 of 17; 95% CI 10–56) in HR-positive/HER2-positive tumors, 71% (5 of 7; 95% CI 29–96) in HR-negative/HER2-positive tumors, and 55% (11 of 20; 95% CI 32–77) in triple-negative tumors (*p* = 0.025).

All characteristics of patients with false-negative biopsies and patients with true-positive biopsies are listed in Table 4. Baseline radiological features (calcifications, multifocality and non-mass) did not differ between the groups. Compared with patients that had true-positive biopsies, patients with false-negative biopsies more often had HR-negative tumors (55% vs 22%, *p* = 0.0006), a higher Bloom-Richardson grade (66% vs 33% grade 3, *p* = 0.006), rCR (90% vs 59%, *p* = 0.005), and less residual invasive disease and/or DCIS in the specimens [6 mm (IQR 3–9) vs 15 mm (IQR 9–29), *p* < 0.001]. The residual disease in patients with false-negative biopsies was more frequently DCIS only (ypTis, 21% vs 4%) than residual invasive disease and DCIS (14% vs 41%) or invasive disease only (65% vs 55%) (*p* = 0.009). In multivariable analysis, only rCR was significantly associated with false-negative biopsies (OR 9.81, 95% CI 1.72–55.89; *p* = 0.01). A trend was seen for HR-negative tumors and smaller size of the residual disease (size in mm) (OR 4.55, 95% CI 0.95–21.73; *p* = 0.058 and OR 0.93, 95% CI 0.87–1.00; *p *= 0.051) (Table 5).

**Table 4:** Characteristics and MICRA assessment in patients with residual disease

	False-negative biopsies (*n* = 29)	True-positive biopsies (*n* = 49)	*P* - value*
*Imaging features pre*-*NST*			
Tumor size (mm)	25 (20–31)	32 (23–58)	0.028
Multifocal	5 (17%)	18 (37%)	0.078
Non-mass	7 (24%)	14 (29%)	0.794
Calcifications	12 (41%)	20 (41%)	1.000
*Histology pre*-*NST*			
Ductal	26 (90%)	39 (80%)	0.423
Lobular	3 (10%)	7 (14%)	
Other	0	3 (6%)	
*Tumor subtype pre*-*NST*			
HR +/HER2 -	8 (28%)	26 (53%)	0.025
HR +/HER2 +	5 (17%)	12 (25%)	
HR -/HER2 +	5 (17%)	2 (4%)	
triple-negative	11 (38%)	9 (18%)	
*Tumor grade pre*-*NST*			
Grade 1	1 (3%)	3 (6%)	0.006
Grade 2	7 (24%)	29 (59%)	
Grade 3	19 (66%)	16 (33%)	
Unknown	2 (7%)	1 (2%)	
**Radiological response**			0.005
Complete	26 (90%)	29 (59%)	
Partial	3 (10%)	20 (41%)	
*Pathology post*-*NST*			
Tumor size (mm)	6 (3–9)	15 (9–29)	<0.001
*DCIS or invasive carcinoma*			
No DCIS	19 (65%)	27 (55%)	0.009
DCIS and invasive	4 (14%)	20 (41%)	
DCIS only	6 (21%)	2 (4%)	

*Kruskal–Wallis rank sum test, Fisher’s exact test. Data are median (IQR) or *n* (%). *NST*, neoadjuvant systemic therapy. All baseline characteristics were assessed before administration of neoadjuvant systemic therapy. Calcifications were assessed on mammography, other imaging features were assessed on MRI

**Table 5:** Predictive factors for false negative MICRA biopsies (*n* = 78)

	Univariable			Multivariable		
	OR	95% CI	*P* - value	OR	95% CI	*P* - value
*Imaging features pre*-*NST*						
Tumor size (mm)	0.98	0.95–1.00	0.066	0.98	0.94–1.01	0.23
Multifocal	0.36	0.12–1.11	0.074			
Non-mass	0.80	0.28–2.28	0.67			
Calcifications	1.02	0.40–2.60	0.96			
*Histology pre*-*NST*						
Ductal	1					
Lobular	0.64	0.15–2.72	0.55			
Other	0.00	0.00–Inf.	0.99			
*HR *≥* 10% pre*-*NST*						
Positive	4.25	1.58–11.48	0.0043	4.55	0.95–21.73	0.058
*Subtype pre*-*NST*						
HR +/HER2 -	1					
HR +/HER2 +	1.35	0.37–5.02	0.65			
HR -/HER2 +	8.12	1.31–50.21	0.024			
triple-negative	3.97	1.21–12.99	0.023			
*Radiological response*						
Partial	1					
Complete	5.98	1.59–22.46	0.008	9.81	1.72–55.89	0.01
*Pathology post*-*NST*						
Tumor size (mm)	0.88	0.81–0.95	0.0006	0.93	0.87–1.00	0.051
*DCIS or invasive carcinoma*						
No DCIS	1					
DCIS and invasive	0.28	0.08–0.97	0.044	0.51	0.12–2.11	0.35
DCIS only	4.26	0.78–23.44	0.095	2.39	0.23–24.37	0.46

Univariable and multivariable logistic regression. *HR*, hormone receptor expression; *NST*, neoadjuvant systemic therapy. All baseline characteristics were assessed before administration of neoadjuvant systemic therapy. Calcifications were assessed on mammography, other imaging features were assessed on MRI

### Adverse Events

Adverse events related to the biopsy procedure were observed in 11 of 167 (7%; 95% CI 3–11) patients. In these patients, the radioactive iodine seed (I-125) used for localization of the tumor area was accidently removed during the biopsy procedure. Removal of the iodine seed led to minor adjustments of the surgical procedure in five patients with planned lumpectomy: in one patient the iodine seed was directly replaced by a new iodine seed, three patients had guided wire localization and in two patients the local excision was widened.

## Discussion

The MICRA trial showed that ultrasound-guided 14-G core biopsies of the breast failed to detect residual disease in approximately one-third of patients with a radiological complete or partial response to NST on DCE-MRI. The MICRA trial was the first trial to study the accuracy of MRI and ultrasound-guided biopsies of the breast after NST to identify pCR of the breast.

Minimally invasive methods aiming to identify patients with pCR of the breast are currently being investigated by several groups.^9^,^14^,^20^ The published literature before this study showed promising results.^20^ In three smaller pilot studies with 20 to 50 patients, FNRs of 5% to 26% were achieved.^9^,^13^,^14^ A larger multicenter exploratory analysis of 164 patients performed by the German Breast Group demonstrated an overall FNR of 49%. In this study, not all patients had a pre-NST placed marker (63%) and biopsy methods were not standardized.^8^ A post hoc analysis in 16 patients with mammographic-guided vacuum-assisted biopsies (VAB) found a FNR of 0%. In the pilot study performed by the University of Heidelberg, the FNR was lowered from 26% to 5% when patients in whom biopsies showed neither tumor cells nor (signs of) the initial tumor bed at histopathological analysis were excluded.^14^ None of these studies used DCE-MRI to select patients with response, as we did in the MICRA trial.

Updated results including a multi-institutional pooled analysis (MDACC, Seoul National University Hospital^21^ and the Royal Marsden Hospital^22^), results of the RESPONDER trial^23^ (NCT02948764, University of Heidelberg), and results of the NRG-BR005 trial^10^ (NRG Oncology) were recently presented.^24^^–^26 The multi-institutional pooled analysis included patients with a partial or complete radiological response on ultrasound, mammography, or MRI, of which 51% had pCR in the surgical specimen.^24^ Vacuum-assisted biopsies (86%) or core-cut biopsies (14%) were performed under ultrasound (78%) or stereotactic (22%) guidance at which a median of six (2–18) 10-G (7–14) biopsies were obtained. The overall FNR was 19% in 159 patients. Post-hoc analysis of patients with a residual imaging abnormality of ≤ 2 cm who had at least six image-guided representative VABs showed a FNR of 3% (*n* = 76).^24^

In the RESPONDER trial^23^, 398 patients were evaluated at interim analysis in which a median of seven 7- to 8-G VABs per patient had been obtained. The FNR was 18%: residual disease was missed in 37 of 208 patients without pCR in the surgical specimen.^25^

The NRG-BR005 trial assessed the accuracy of six to eight 11-G biopsies in patients with ductal carcinoma and a clinical (near) complete response with tri-modality imaging after NST: < 1-cm residual mass on mammography (no calcifications), < 2-cm residual mass on ultrasound, no rapid rise or washout kinetics on a 1.5-T post-NST MRI.^10^ At the planned interim analysis, 36 out of 98 evaluable patients had residual disease at surgery, of which 18 patients were not correctly identified by post-NST biopsies (FNR of 50%).^26^

Compared with the RESPONDER trial and the multi-institutional pooled analysis, we found a relatively high FNR for biopsies detecting residual disease. Key differences in the study designs were patient selection criteria and biopsy technique. The MICRA trial and the NRG-BR005 trial were the only trials that used DCE-MRI to select patients with therapy response. The NRG-BR005 trial, however, only assessed therapy response on post-NST MRI, whereas both pre- and post-NST MR-images were used in the MICRA trial for adequate response evaluation. As DCE-MRI is more accurate in selecting patients with a (near) pCR compared with conventional imaging, the proportion of patients with substantial residual disease in the studies that used conventional imaging for response monitoring might be higher, which will lower the reported FNR.

We found a significantly higher FNR in patients with no rCR on MRI than in patients with residual enhancement (47% vs 13%). Patients with false-negative biopsies had less residual disease in the surgical specimens than patients with true-positive biopsies, and tumors were more often triple-negative and HR-negative/HER2-positive, which are the subtypes that respond well to NST. Hence, these factors that are predictive for a false-negative outcome represent the same causal mechanism: sampling errors occur more frequently in patients with minimal residual disease after NST.

The results the MICRA trial and those of the previous studies emphasize that current imaging modalities, including MRI, are not accurate enough to identify patients with pCR for omission of surgery.^6^,^7^ We found residual disease in the surgical specimens of 40% of patients with rCR. In the patients with rPR, 26% did achieve pCR at time of surgery.

One major difference between the previous studies mentioned and the MICRA study is the quantity of tissue obtained and examined with biopsies. In the MICRA trial core biopsies were performed, whereas vacuum-assisted biopsies were used in most other trials. With 9-G to 10-G vacuum-assisted biopsies, approximately 7 times as much tissue per biopsy is obtained compared with 14-G core needle biopsies, making assessment more reliable.^27^,^28^ However, VAB procedures are also associated with more patient discomfort and may be associated with more severe bleeding events.^29^

Another limitation of the MICRA trial was that all biopsies were obtained immediately before breast surgery in the operating room, with the patient under general anesthesia. This procedure minimized patient discomfort, but most likely affected the accuracy of the biopsies. The ultrasound equipment used for the biopsy procedure in the operating room was sometimes inferior to that of the radiology department. Optimal positioning of the patient under general anesthesia in an operating room was more difficult compared with the normal setting in the radiology department, resulting in more difficult biopsy angles. However, biopsies were not performed if the marker could not be visualized during the procedure (21 patients) and parts of the (former) tumor area were seen in at least one of the biopsies obtained in almost all patients.

In 89% of all patients, at least eight biopsies could be obtained. Only six (4%) patients underwent fewer than six biopsies. Representativeness of the biopsies was marked as “unknown” (i.e., sufficient material for analysis, but no signs of therapy response or tumor) in eight (5%) patients. In four of these patients, residual disease was found in the surgical specimen. Another eight patients were found to have insufficient biopsy specimens for a pathological diagnosis, of which two patients had residual disease. Excluding these patients from the analysis, however, would not have resulted in a significantly improved FNR (32% vs 37%).

The ultimate aim of the MICRA trial was to develop an accurate minimally invasive method that would identify pCR in patients with a radiological response and thereby potentially allow omission of surgery of the breast in these patients. At the same time, it is important to accurately identify patients who do not achieve pCR, as patients with residual disease after NST have a significantly worse prognosis and may benefit from additional systemic treatment.^30^^–^32 In addition, although the correlation is strong, pCR of the breast (ypT0) does not entirely exclude the presence of lymph node metastases (ypN +).^33^ Several studies are currently investiagting the de-escalation of axillary surgery after NST.^34^,^35^ If breast surgery after NST in patients with pCR could be omitted in the near future, simulteneous de-escalation of axillary surgery will be essential.

The optimal cut-off value for the FNR of biopsies (and type and extent of the errors) identifying pCR for a clinically acceptable recurrence rate, is yet unknown. Investigators from the MDACC have already started a trial (NCT02945579) in which breast surgery is omitted in early stage triple-negative or HER2-positive breast cancer patients who have at least 12 tumor-negative VABs. The primary outcome is 5-year locoregional recurrence-free survival.^20^

Although the minimally invasive method developed in the MICRA trial may not be used for omission of surgery, the interim results contribute to the development of more accurate methods for detection of pCR in patients with an excellent response on MRI after NST. The risk of sampling errors in patients who are most likely to have limited residual disease after NST may be reduced by obtaining larger, vacuum-assisted biopsies under optimal conditions in the radiology department. The development of non-invasive response prediction models incorporating biomarkers and MRI radiomics using machine-learning, on the other hand, may eventually outperform minimally invasive pCR detection methods. Regardless of the methods used to identify pCR, it will be essential to decide to what extent a possibly increased risk of local recurrence outweighs the benefits of elimination of breast surgery. We will continue to investigate minimally invasive techniques predicting pCR to ultimately achieve an operation-free treatment strategy for patients with pCR after NST.

## Electronic supplementary material

Below is the link to the electronic supplementary material.

Supplementary material 1 (DOCX 18 kb)
