# Supplementary material for: Minimally Invasive Complete Response Assessment of the Breast After Neoadjuvant Systemic Therapy for Early Breast Cancer (MICRA trial): Interim Analysis of a Multicenter Observational Cohort Study
Source: Ann Surg Oncol. 2020 Dec 2;28(6):3243–53. doi: 10.1245/s10434-020-09273-0 (PMC8119397; doi:10.1245/s10434-020-09273-0)
Supplement: Supplementary file 1 — Supplementary material 1 (DOCX 18 kb) [file 10434_2020_9273_MOESM1_ESM.docx]

**SUPPLEMENTARY APPENDIX**

Supplement to: ‘Minimally Invasive Complete Response Assessment of the breast after neoadjuvant systemic therapy for early breast cancer (MICRA trial): interim analysis of a multicenter observational cohort study’

**Content**

Supplementary table

| **Supplementary table 1.**  Completed and ongoing trials investigating the accuracy of biopsies identifying a pathological complete response of the breast. | | | | | | | |
| --- | --- | --- | --- | --- | --- | --- | --- |
| **Study/Trial** | **Start date** | **Tumour subtype** | **Response**  **evaluation** | **Biopsy method** | **No. of patients** | **Results/**  **outcome** | **End date** |
| **German Breast group**  Heil et al. | Dec 2009  Retrospective | All | cCR at PE or MG/US/MRI | 9–11G VAB or 14G CC | 164 | FNR 49.3%  (35/71) | Dec 2013 |
| **NOSTRA PRELIM**  Rea-Francis et al | Oct 2014 | HER2+ & TN | None | 2–6x USG  CC | 20 | FNR 22%  (4/18) | Apr 2016 |
| **University of Heidelberg**  Heil et al.  NCT02575612 | Jul 2014 | All | rPR or rCR on  MG or US/ MRI | 6–12x USG  9G VAB | 50 | FNR 25.9%  (7/27) | Feb 2015 |
| **RESPONDER**  Heil et al.  NCT02948764 | Mar 2015 | All | rPR or rCR on  MG or US/MRI | 7x USG/ STX  7-10G VAB | 398 | FNR 18%  (37/208) | Jun 2019  Suspended |
| **MD Anderson Cancer Centre**. Kuerer et al.  NCT02455791 | Jun 2015 | HER2+ & TN | <5cm | 12x USG/STX  9G VAB and FNA | 40 | FNR 5%  (1/21) | Dec 2016 |
| **MICRA trial**  Netherlands Cancer Institute  NTR6120 | Apr 2016 | All | rPR or rCR on MRI; ≤2 cm | 8x USG  14G CC | 167 | FNR 37%  (29/49) | Jun 2019  Suspended |
| **Seoul National University Hospital**  NCT03273426 | Sep 2016 | All | rCR/near-rCR on MRI | ≥ 5 USG  14G CC or 10G VAB | 40 | FNR 30.8%  (4/13) | Dec 2017 |
| **MD Anderson Cancer Centre**. Kuerer et al.  NCT02945579 | Jan 2017 | HER2+ & TN | <5 cm and final size  < 2 cm | 12x USG/STX  9G VAB | 50 | 5y LRR < 7% | Ongoing |
| **NRG Oncology**  **BR005**  NCT03188393 | Apr 2017 | All | rCR/near-rCR on MG or US/ MRI | 6x STX  8-11G VAB | 98 | FNR 50%  (18/36) | Aug 2019  Suspended |
| **Memorial Sloan Kettering**  **Cancer centre**  NCT03289195 | Sep 2017 | All | rCR on MRI | MRI-guided biopsy | 25 | NPV | Ongoing |
| **The Royal Marsden NHS Foundation**  Tasoulis et al. | Jan 2013  Retrospective | All | rPR or rCR | USG/ STX  VAB | 53 | FNR 19%  (6/31) | Jan 2018 |
| **The Royal Marsden NHS Foundation**  Teoh et al. | Feb 2018 | HER2+ & TN | rPR or rCR  ≤ 2cm | USG/ STX  VAB | 28 | FNR 9%  (1/11) | June 2019 |
| **Basavatarakam Indo American Cancer Institute**  Hydarabad  CTRI/2018/01/011122 | May 2019 | All | rCR/near-rCR on MG/US | 4x USG  14G CC | 65 | FNR 14.8%  (x/38) | Nov 2019 |
| **NeoVAB**  Centre Georges Francois Leclerc  NCT03876951 | Jun 2019 | All | cCR at PE or MG/US/MRI | USG/STX  VAB | 66 | FNR | Ongoing |
| **Masonic Cancer Centre**  University of Minnesota  NCT03981705 | Aug 2019 | HER2+ & TN | - | - | 40 | FNR | Ongoing |
| HER2+ = human epidermal growth factor 2- positive, TN = triple negative, cCR = clinical complete response, rCR = radiological complete response, rPR = radiological partial response, PE = physical examination, MG = mammogram, US = ultrasound, MRI = magnetic resonance imaging, G = gauge, VAB = vacuum assisted biopsy, CC = core cut biopsy, USG = ultrasound-guided STX = stereotactic, FNA = fine-needle aspiration, FNR = false-negative rate. | | | | | | | |
